# Cerebral Oxygenation Changes During Sequential Postural Transitions From Supine to Walking in Healthy Adults: An Exploratory Near-Infrared Spectroscopy Study

**DOI:** 10.7759/cureus.115581

**Published:** 2026-09-01

**Authors:** Kenji Oike, Atsuhiro Tsubaki, Tetsuya Takahashi

**Affiliations:** 1 Rehabilitation, Tsuchiura Kyodo General Hospital, Tsuchiura, JPN; 2 Physical Therapy, Graduate School of Health Sciences, Juntendo University, Tokyo, JPN; 3 Physical Medicine and Rehabilitation, Institute for Human Movement and Medical Sciences, Niigata University of Health and Welfare, Niigata, JPN

**Keywords:** cerebral oxygenation, near-infrared spectroscopy, orthostatic stress, postural change, walking

## Abstract

Objective

Early mobilization (EM) in the intensive care unit (ICU) reduces complications from prolonged bed rest and improves functional outcomes. However, there are still no objective criteria for cerebral hemodynamics during postural changes, especially walking.

Methods

This exploratory observational study used near-infrared spectroscopy (NIRS) to measure prefrontal cerebral oxygenation changes in 14 healthy adults during sequential postural transitions from supine rest to sitting, standing, and walking. The following parameters were assessed: oxygenated hemoglobin (O₂Hb), deoxygenated hemoglobin (HHb), total hemoglobin (tHb), regional cerebral oxygen saturation (rSO₂), mean arterial pressure (MAP), and heart rate (HR).

Results

ΔO₂Hb decreased, and ΔHHb increased relative to the supine baseline, with significant differences in the magnitude of change across sitting, standing, and walking (ΔO₂Hb, p = 0.012; ΔHHb, p < 0.001). rSO₂ differed significantly across the four postural conditions, with lower values observed in upright postures than in the supine position (p < 0.001). The magnitude of ΔtHb did not differ significantly among sitting, standing, and walking (p = 0.257). MAP (p = 0.015) and HR (p < 0.001) differed significantly across the four postural conditions.

Conclusions

Sequential postural transitions from supine rest to sitting, standing, and walking were associated with changes in NIRS-derived cerebral oxygenation in healthy adults, characterized by decreases in O₂Hb and increases in HHb during progression to upright postures. These findings characterize cerebral oxygenation changes across a stepwise mobilization sequence and may provide preliminary reference data for evaluating cerebral oxygenation during mobilization.

## Introduction

Early mobilization (EM) is an important component of early rehabilitation in the intensive care unit (ICU) and may reduce complications associated with prolonged bed rest and improve functional outcomes [1,2]. The safety and efficacy of EM have been established across diverse patient populations, with guidelines recommending structured protocols that include start and stop criteria [3-5]. These criteria typically incorporate objective vital signs, such as blood pressure, heart rate (HR), and percutaneous arterial oxygen saturation (SpO₂). Neurological assessments, including the Glasgow Coma Scale (GCS), may also be used to assess consciousness and neurological status [6,7]; however, they do not directly assess cerebral hemodynamics or oxygenation and are not routinely incorporated into formal EM start/stop criteria [3-5]. Thus, conventional clinical assessments provide limited information regarding cerebral physiological responses during mobilization, highlighting the need for objective measures that can complement neurological and systemic assessments.

Walking differs from static sitting and standing because it combines an upright posture with dynamic whole-body activity and additional cardiovascular and metabolic demands. These characteristics may result in cerebral oxygenation responses that differ from those observed during static postural conditions, providing a physiological rationale for evaluating walking separately within a sequential mobilization protocol.

Traditionally, cerebral autoregulation refers to maintaining stable blood flow across a range of steady blood pressures (static autoregulation) [8]. Recently, however, dynamic autoregulation has been considered crucial for maintaining cerebral perfusion during changes in posture and activity, though its clinical significance remains to be studied further [9,10]. Near-infrared spectroscopy (NIRS) provides a noninvasive means of monitoring cerebral oxygenation [11] and enables real-time evaluation of hemodynamic responses across various physiological states [12]. Despite its limited penetration depth, NIRS offers unique insights into cortical hemodynamics during dynamic tasks. Emerging studies have used NIRS to examine cerebral oxygenation dynamics during EM in critically ill patients [13], heart failure patients [14], and stroke survivors [15,16].

Nevertheless, reference data on NIRS-derived cerebral oxygenation measures, including O₂Hb, HHb, tHb, and rSO₂, during standardized sequential mobilization from supine rest through sitting and standing to walking remain limited [17,18]. Without such reference data, distinguishing physiological cerebral oxygenation responses during mobilization from potentially abnormal responses in clinical populations remains challenging.

The aim of this exploratory study was to characterize prefrontal cerebral oxygenation changes during a sequential mobilization protocol from supine rest through sitting and standing to walking in healthy adults using NIRS. Characterizing these physiological responses may provide preliminary reference data for interpreting NIRS-derived cerebral oxygenation changes during mobilization.

This work was previously presented as an oral presentation at the 31st Meeting of the Medical Near-Infrared Spectroscopy Research Society in September 2025.

## Materials and methods

Study design and participants

This prospective observational study was conducted at Tsuchiura Kyodo General Hospital, Tsuchiura, Ibaraki, Japan, from August 2024 to January 2025. Healthy adult volunteers from our institution were recruited. The inclusion criteria were adults with no medical abnormalities identified during recent routine health checkups, who were able to independently perform the sequential postural protocol consisting of supine rest, sitting, standing, and walking, and who provided written informed consent to participate in the study. The exclusion criteria were a self-reported history of cerebrovascular disease, neurological disease, orthopedic disease that could interfere with the postural protocol, cardiac disease, or diabetes mellitus; current medication use considered potentially relevant to cardiovascular or cerebrovascular responses; or subjective symptoms that could interfere with safe completion of the postural protocol.

At enrollment, participants were screened using a study-specific, non-validated screening form developed by the investigators to collect information on medical history, including cerebrovascular, neurological, orthopedic, and cardiac diseases and diabetes mellitus, current medication use, and subjective symptoms. This form was used solely for eligibility screening and was not a standardized or validated questionnaire. None of the enrolled participants met the exclusion criteria. Written informed consent was obtained from all participants. The study was approved by the Ethics Committee of Tsuchiura Kyodo General Hospital (approval number: 2024FY87). All procedures were conducted in accordance with the Declaration of Helsinki and relevant institutional and national guidelines and regulations.

Cerebral oxygenation measurement

Cerebral oxygenation was continuously monitored using a two-channel NIRS device (Brain Activity Monitor Hb134; Astem, Kanagawa, Japan). Probes were placed bilaterally over the prefrontal cortex at Fp1 and Fp2 according to the international 10-20 system [19,20]. Oxygenated hemoglobin (O₂Hb), deoxygenated hemoglobin (HHb), total hemoglobin (tHb), and regional cerebral oxygen saturation (rSO₂) were continuously recorded at a sampling frequency of 10 Hz throughout the protocol.

Postural protocol

Following approximately 10 minutes of supine rest, participants underwent a sequential postural protocol consisting of sitting, standing, and walking (Figure 1) [21].

**Figure 1:**
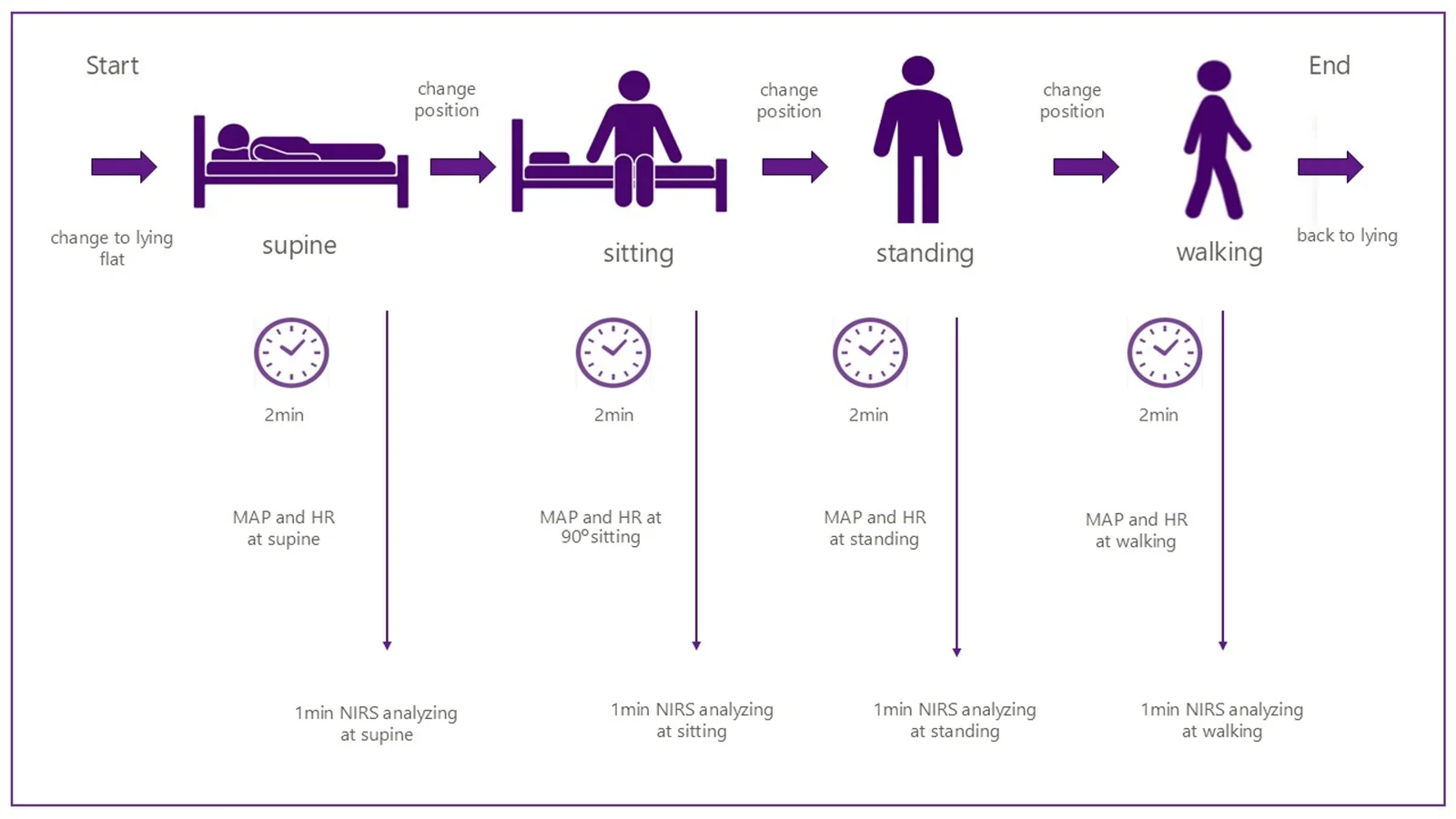
Study protocol for sequential postural transitions and physiological measurements Figure 1 shows the experimental protocol. Participants changed posture every two minutes: supine, sitting, standing, and walking. MAP and HR were measured, and NIRS analysis was performed during the last minute of each posture. Walking was performed at a self-selected comfortable speed. Image credits: Kenji Oike. No artificial intelligence (AI) tools were used to generate or modify this figure. MAP: Mean arterial pressure; HR: Heart rate; NIRS: Near-infrared spectroscopy.

The sequence was fixed as supine, sitting, standing, and walking to reflect the stepwise progression used during mobilization. Therefore, the protocol was intended to evaluate cerebral oxygenation changes across a sequential mobilization progression rather than the independent effects of each condition. The final 60 seconds of the supine resting period were averaged and used as the baseline. Each sitting and standing condition and the walking period lasted for 2 minutes to allow an initial period of physiological adjustment after each transition. NIRS data from the final 60 seconds of each condition were averaged to reduce the influence of the immediate transitional response and to obtain a representative value for each condition. Participants independently assumed sitting and standing positions without assistance. Walking was performed at an individually preferred pace. All measurements were performed in a dimly lit, temperature-controlled room (22°C-25°C) between 5:00 p.m. and 6:00 p.m. to standardize measurement conditions.

Parameters and data analysis

NIRS signals were visually inspected, and segments containing obvious motion artefacts were excluded from the analysis. Signals obtained from the left and right prefrontal regions were averaged for analysis. Outcome variables included O₂Hb, HHb, tHb, and rSO₂. Changes from the supine baseline (ΔO₂Hb, ΔHHb, and ΔtHb) were calculated for each postural condition.

Cardiovascular parameters

Mean arterial pressure (MAP) and HR were measured using an iLife Scope PT patient monitor (Nihon Kohden, Tokyo, Japan). MAP was measured once during the final minute of the supine, sitting, and standing conditions using an automated non-invasive upper-arm cuff. For the walking condition, MAP was measured immediately after completion of the walking period. HR was continuously monitored using three-lead electrocardiography (Lead II) with electrodes placed on the chest. For analysis, HR values from the final minute of each posture were used to align with the NIRS analysis period.

Sample size

An a priori power analysis was performed using G*Power (version 3.1; Heinrich-Heine-Universität Düsseldorf, Düsseldorf, Germany) [22,23]. As directly comparable effect-size estimates for cerebral oxygenation changes across the present sequential postural protocol were unavailable, a moderate effect size (f = 0.30) was assumed for planning purposes, with reference to previous studies demonstrating measurable cerebral oxygenation changes during postural transitions in healthy adults. A correlation of 0.70 among repeated measures was used as a planning assumption because repeated measurements were obtained within the same participants under standardized conditions; an empirical correlation estimate specific to the present protocol was not available. With a power of 0.80, an alpha level of 0.05, and a nonsphericity correction (ε) of 0.8, the required sample size was estimated to be 13 participants. Ultimately, 14 participants were enrolled.

Statistical analysis

Continuous variables are presented as mean ± standard deviation (SD), and categorical variables are presented as number and percentage. For O₂Hb, HHb, and tHb, changes from the supine baseline (ΔO₂Hb, ΔHHb, and ΔtHb, respectively) were calculated for sitting, standing, and walking. The three change scores (supine-to-sitting, supine-to-standing, and supine-to-walking) were compared using the Friedman test. When a significant overall difference was detected, pairwise comparisons among the three change scores were performed using Wilcoxon signed-rank tests with Bonferroni correction for multiple comparisons. Kendall’s W was calculated as an effect-size measure for each Friedman test. For rSO₂, HR, and MAP, absolute values were compared across the four postural conditions (supine, sitting, standing, and walking) using the Friedman test. When a significant overall difference was detected, pairwise comparisons among the four postural conditions were performed using Wilcoxon signed-rank tests with Bonferroni correction for multiple comparisons. All statistical tests were two-sided, and a p-value < 0.05 was considered statistically significant. Statistical analyses were performed using EZR (version 1.52; Saitama Medical Center, Jichi Medical University, Saitama, Japan) [24].

## Results

Fourteen healthy adults (five females and nine males; mean age, 35.4 ± 7.8 years) participated in the study (Table 1).

**Table 1:** Participant characteristics Data are presented as mean ± standard deviation or n (%), as appropriate. SpO₂: Peripheral oxygen saturation.

Characteristics	Value
Age, years	35.4 ± 7.8
Female, n (%)	5 (35.7)
Male, n (%)	9 (64.3)
Resting systolic blood pressure, mmHg	112.6 ± 13.5
Resting diastolic blood pressure, mmHg	65.9 ± 10.1
Resting SpO₂, %	98.1 ± 0.7

Table 2 summarizes changes in O₂Hb, HHb, and tHb from the supine baseline (ΔO₂Hb, ΔHHb, and ΔtHb, respectively).

**Table 2:** Changes in cerebral oxygenation parameters during postural transitions from supine to sitting, standing, and walking Values are presented as mean ± SD. Δ values represent changes from the supine baseline. Overall comparisons were performed using the Friedman test. Pairwise comparisons were performed using Wilcoxon signed-rank tests with Bonferroni correction; significant pairwise comparisons are reported in the text. O₂Hb: Oxygenated hemoglobin; HHb: Deoxygenated hemoglobin; tHb: Total hemoglobin; SD: Standard deviation.

Parameters	Supine to sitting	Supine to standing	Supine to walking	Friedman χ² (2)	p-value
ΔO₂Hb (μmol/L)	-17.9 ± 23.6	-23.6 ± 26.6	-26.6 ± 25.0	8.71	0.012
ΔHHb (μmol/L)	10.1 ± 16.5	13.4 ± 16.3	15.6 ± 14.6	16.71	<0.001
ΔtHb (μmol/L)	-7.8 ± 7.2	-10.2 ± 10.1	-11.0 ± 9.8	2.71	0.257

ΔO₂Hb, representing the change from the supine baseline, was -17.9 ± 23.6 μmol/L for sitting, -23.6 ± 26.6 μmol/L for standing, and -26.6 ± 25.0 μmol/L for walking. The magnitude of change differed significantly across the three postural conditions (χ² (2) = 8.71, p = 0.012, Kendall’s W = 0.31). Bonferroni-corrected post hoc comparisons showed that the decrease in O₂Hb from the supine baseline was significantly greater for standing than for sitting (p = 0.04) and for walking than for sitting (p = 0.04), whereas the magnitude of the decrease did not differ significantly between standing and walking (p = 1.00).

ΔHHb, representing the change from the supine baseline, was 10.1 ± 16.5 μmol/L for sitting, 13.4 ± 16.3 μmol/L for standing, and 15.6 ± 14.6 μmol/L for walking. The magnitude of change differed significantly across the three postural conditions (χ² (2) = 16.71, p < 0.001, Kendall’s W = 0.60). Bonferroni-corrected post hoc comparisons showed that the increase in HHb from the supine baseline was significantly greater for standing than for sitting (p < 0.001) and for walking than for sitting (p = 0.007), whereas the magnitude of the increase did not differ significantly between standing and walking (p = 0.126).

ΔtHb, representing the change from the supine baseline, was -7.8 ± 7.2 μmol/L for sitting, -10.2 ± 10.1 μmol/L for standing, and -11.0 ± 9.8 μmol/L for walking. The magnitude of change did not differ significantly across the three postural conditions (χ² (2) = 2.71, p = 0.257). The magnitude of ΔtHb did not differ significantly among the sitting, standing, and walking conditions (χ² (2) = 2.71, p = 0.257, Kendall’s W = 0.10).

Table 3 presents absolute values of rSO₂, HR, and MAP across the four postural conditions.

**Table 3:** rSO₂, MAP, and HR across postural transitions from supine to sitting, standing, and walking Values are presented as mean ± SD. Overall comparisons were performed using the Friedman test. Pairwise comparisons were performed using Wilcoxon signed-rank tests with Bonferroni correction; significant pairwise comparisons are reported in the text. rSO₂: Regional cerebral oxygen saturation; MAP: Mean arterial pressure; HR: Heart rate; SD: Standard deviation.

Parameters	Supine	Sitting	Standing	Walking	Friedman χ² (3)	p-value
rSO₂ (%)	66.0 ± 2.1	64.5 ± 2.6	62.6 ± 2.3	62.1 ± 2.3	21.86	<0.001
MAP (mmHg)	81.4 ± 10.5	84.4 ± 10.9	87.5 ± 9.9	85.1 ± 10.8	10.51	0.015
HR (bpm)	64.6 ± 7.7	71.9 ± 9.6	78.6 ± 11.4	78.0 ± 13.0	17.59	<0.001

rSO₂ differed significantly across the four postural conditions (supine, 66.0 ± 2.1%; sitting, 64.5 ± 2.6%; standing, 62.6 ± 2.3%; walking, 62.1 ± 2.3%; χ² (3) = 21.86, p < 0.001, Kendall’s W = 0.52). Post hoc analyses revealed significant differences between the supine and sitting (p = 0.007) positions, the sitting and standing (p = 0.005) positions, and the supine and standing (p = 0.001) positions.

MAP differed significantly across the four postural conditions (supine, 81.4 ± 10.5 mmHg; sitting, 84.4 ± 10.9 mmHg; standing, 87.5 ± 9.9 mmHg; walking, 85.1 ± 10.8 mmHg; χ² (3) = 10.51, p = 0.015, Kendall’s W = 0.25). Post hoc analyses revealed a significant difference between the supine and standing conditions (p = 0.013); other comparisons were not significant.

HR differed significantly across the four conditions (average: supine 64.6 ± 7.7 beats per minute [bpm], sitting 71.9 ± 9.6 bpm, standing 78.6 ± 11.4 bpm, walking 78.0 ± 13.0 bpm; Friedman test, χ² (3) = 17.59, p < 0.001, Kendall’s W = 0.42). Post hoc tests showed that the supine condition differed from the sitting (p = 0.045), standing (p = 0.010), and walking (p = 0.013) conditions; other comparisons were not significant.

## Discussion

This exploratory study examined prefrontal cerebral oxygenation changes during sequential postural transitions from a supine position to walking in healthy adults using NIRS. The magnitude of the decrease in O₂Hb and increase in HHb from the supine baseline differed significantly across sitting, standing, and walking (O₂Hb, p = 0.012; HHb, p < 0.001), whereas the magnitude of change in tHb did not differ significantly (p = 0.257). rSO₂ differed significantly across the four postural conditions, with lower values observed in upright postures than in the supine position (p < 0.001). MAP also differed significantly across postural conditions (p = 0.015), with a significant increase from supine to standing. HR differed significantly across postural conditions (p < 0.001), with higher values in sitting, standing, and walking than in the supine position.

A distinctive feature of the present study was the evaluation of cerebral oxygenation across the entire stepwise progression from supine rest to sitting, standing, and walking within the same participants. This approach provides insight into cerebral oxygenation changes during sequential postural transitions, including the transition to walking. These findings align with prior reports in healthy individuals, in which O₂Hb decreases and HHb increases in upright postures compared to the supine position, with reductions in rSO₂ and stable MAP [17,18,25,26].

Notably, the decrease in O₂Hb and increase in HHb from the supine baseline were greater during standing and walking than during sitting, whereas no significant differences were observed between standing and walking. However, the absence of statistically significant differences between standing and walking should not be interpreted as evidence of equivalence between these conditions, particularly given the small sample size. While previous studies often focused on supine-to-standing comparisons [17,18], the present study extends these observations by examining the full sequential progression to walking.

Physiologically, the observed decrease in O₂Hb and increase in HHb indicate changes in NIRS-derived cerebral oxygenation signals during the transition to upright postures. Previous studies have shown that orthostatic stress causes gravitational pooling of blood in the lower extremities and reduces venous return to the heart [17, 27], which may alter cerebral perfusion and oxygen delivery [17,26,27]. Thus, the NIRS changes observed in the present study may be consistent with cerebral oxygenation responses to orthostatic stress. However, cerebral blood flow was not directly measured, and NIRS-derived O₂Hb, HHb, and rSO₂ may also be influenced by changes in arterial and venous blood volume, extracerebral tissue perfusion, probe-related factors, and systemic hemodynamics. Therefore, the underlying physiological mechanisms cannot be determined from the present NIRS findings alone.

The magnitude of ΔtHb did not differ significantly among sitting, standing, and walking. Importantly, this finding does not indicate that tHb remained unchanged from the supine baseline or that cerebral blood volume remained stable. Although a previous study found no significant differences in tHb between slow and rapid supine-to-stand transitions despite changes in O₂Hb and HHb [17], direct comparison with the present sequential protocol should be made cautiously.

HR and MAP differed significantly across postural conditions, consistent with systemic cardiovascular responses to postural change. These findings are consistent with previous studies demonstrating cardiovascular compensatory responses during orthostatic stress in healthy individuals [25-27]. However, MAP was measured intermittently, and the walking MAP measurement was obtained immediately after completion of walking. Therefore, transient beat-to-beat blood pressure changes could not be assessed, and the present measurements did not permit formal assessment of dynamic cerebral autoregulation [9]. Accordingly, no conclusions regarding dynamic cerebral autoregulatory function can be drawn from the present data.

Since this study focused on healthy adults, the observed responses may provide preliminary reference data for physiological cerebral oxygenation changes during sequential postural transitions. However, further research is needed to understand the mechanisms that regulate regional cerebral blood flow during posture changes. These methods show potential for clinically assessing cerebral circulation during EM in ICU patients or those with neurological disorders, which are future research areas. Future studies should determine whether deviations from the cerebral oxygenation responses observed in healthy adults are associated with clinically relevant cerebral hemodynamic disturbances during mobilization in critically ill or neurological populations.

Limitations

This study has several limitations. First, the sample size was small (n = 14), limiting the precision of the estimates and the ability to detect potentially meaningful differences, particularly for nonsignificant comparisons. Therefore, the absence of statistical significance should not be interpreted as evidence of equivalence between conditions. Second, MAP was measured intermittently, and the walking MAP measurement was obtained immediately after walking; therefore, transient hemodynamic responses and dynamic cerebral autoregulation could not be assessed. Third, NIRS-derived measures are indirect and may be influenced by extracerebral tissue perfusion, probe-related factors, systemic hemodynamics, and motion artefacts. Although NIRS signals were visually inspected and segments containing obvious motion artefacts were excluded, residual motion-related effects, particularly during walking, cannot be excluded. As cerebral blood flow was not directly measured, the physiological mechanisms underlying the observed NIRS changes cannot be determined from the present data. Fourth, because all participants underwent the postural conditions in a fixed sequence, potential order or time effects cannot be excluded. The findings should therefore be interpreted as responses during the sequential mobilization protocol rather than as the independent effects of each posture. Finally, the inclusion of young, healthy participants limits generalizability to older adults and clinical populations.

## Conclusions

This exploratory study characterized prefrontal cerebral oxygenation changes during sequential postural transitions from supine rest to sitting, standing, and walking in healthy adults using NIRS. O₂Hb decreased and HHb increased during the progression to upright postures, whereas the magnitude of ΔtHb did not differ significantly among sitting, standing, and walking. No statistically significant differences in O₂Hb or HHb changes were detected between standing and walking. Importantly, the present study demonstrates that NIRS can characterize cerebral oxygenation changes across the entire stepwise mobilization sequence within the same participants. These findings provide preliminary reference data for evaluating cerebral oxygenation during sequential mobilization. Given the small sample size and indirect nature of NIRS measurements, the underlying physiological mechanisms should be interpreted cautiously.

## References

[1] Schweickert WD, Pohlman MC, Pohlman AS (2009). Early physical and occupational therapy in mechanically ventilated, critically ill patients: a randomised controlled trial. Lancet.

[2] Tipping CJ, Harrold M, Holland A, Romero L, Nisbet T, Hodgson CL (2017). The effects of active mobilisation and rehabilitation in ICU on mortality and function: a systematic review. Intensive Care Med.

[3] Lewis K, Balas MC, Stollings JL (2025). A Focused Update to the Clinical Practice Guidelines for the prevention and management of pain, anxiety, agitation/sedation, delirium, immobility, and sleep disruption in adult patients in the ICU. Crit Care Med.

[4] Unoki T, Hayashida K, Kawai Y (2023). Japanese Clinical Practice Guidelines for rehabilitation in critically Ill patients 2023 (J-ReCIP 2023). J Intensive Care.

[5] Hodgson CL, Stiller K, Needham DM (2014). Expert consensus and recommendations on safety criteria for active mobilization of mechanically ventilated critically ill adults. Crit Care.

[6] Takahashi T, Kato M, Obata K (2021). Minimum standards of clinical practice for physical therapists working in intensive care units in Japan. Phys Ther Res.

[7] Skinner EH, Thomas P, Reeve JC, Patman S (2016). Minimum standards of clinical practice for physiotherapists working in critical care settings in Australia and New Zealand: a modified Delphi technique. Physiother Theory Pract.

[8] Paulson OB, Strandgaard S, Edvinsson L (1990). Cerebral autoregulation. Cerebrovasc Brain Metab Rev.

[9] Elting JW, Sanders ML, Panerai RB (2020). Assessment of dynamic cerebral autoregulation in humans: is reproducibility dependent on blood pressure variability?. PLoS One.

[10] Aaslid R, Lindegaard KF, Sorteberg W, Nornes H (1989). Cerebral autoregulation dynamics in humans. Stroke.

[11] Jöbsis FF (1977). Noninvasive, infrared monitoring of cerebral and myocardial oxygen sufficiency and circulatory parameters. Science.

[12] Villringer A, Planck J, Hock C, Schleinkofer L, Dirnagl U (1993). Near infrared spectroscopy (NIRS): a new tool to study hemodynamic changes during activation of brain function in human adults. Neurosci Lett.

[13] Imai R, Abe T, Iwata K, Yamaguchi S, Kitai T, Tsubaki A (2025). Regional cerebral oxygen saturation during initial mobilization of critically ill patients is associated with clinical outcomes: a prospective observational study. Intensive Care Med Exp.

[14] Imai R, Abe T, Yamaguchi S, Kimura S, Tsubaki A (2024). Relationship between regional cerebral oxygen saturation and percutaneous oxygen saturation at initial mobilisation in patients with acute heart failure. Adv Exp Med Biol.

[15] Maekawa Y, Iwata K, Inagaki Y, Takahashi T, Yamada K, Kohara N, Tsubaki A (2024). Effects of head elevation on cerebral oxygenation in acute cerebral infarction. Adv Exp Med Biol.

[16] Moriya M (2022). Changes in prefrontal cortical oxygenation during tilt table orthostatic hypotension in subarachnoid haemorrhage patients. Adv Exp Med Biol.

[17] Mol A, Woltering JH, Colier WN, Maier AB, Meskers CG, van Wezel RJ (2019). Sensitivity and reliability of cerebral oxygenation responses to postural changes measured with near-infrared spectroscopy. Eur J Appl Physiol.

[18] Mol A, Claassen JA, Maier AB, van Wezel RJ, Meskers CG (2022). Determinants of orthostatic cerebral oxygenation assessed using near-infrared spectroscopy. Auton Neurosci.

[19] Niwayama M (2018). Voxel-based measurement sensitivity of spatially resolved near-infrared spectroscopy in layered tissues. J Biomed Opt.

[20] Klem GH, Lüders HO, Jasper HH, Elger C (1999). The ten-twenty electrode system of the International Federation. The International Federation of Clinical Neurophysiology. Electroencephalogr Clin Neurophysiol Suppl.

[21] Carvalho LB, Kaffenberger T, Chambers B (2024). Cerebral hemodynamic response to upright position in acute ischemic stroke. Front Neurol.

[22] Faul F, Erdfelder E, Lang AG, Buchner A (2007). G*Power 3: a flexible statistical power analysis program for the social, behavioral, and biomedical sciences. Behav Res Methods.

[23] Faul F, Erdfelder E, Buchner A, Lang AG (2009). Statistical power analyses using G*Power 3.1: tests for correlation and regression analyses. Behav Res Methods.

[24] Kanda Y (2013). Investigation of the freely available easy-to-use software 'EZR' for medical statistics. Bone Marrow Transplant.

[25] van Lieshout JJ, Pott F, Madsen PL, van Goudoever J, Secher NH (2001). Muscle tensing during standing: effects on cerebral tissue oxygenation and cerebral artery blood velocity. Stroke.

[26] Kim YS, Bogert LW, Immink RV, Harms MP, Colier WN, van Lieshout JJ (2011). Effects of aging on the cerebrovascular orthostatic response. Neurobiol Aging.

[27] Jacob G, Ertl AC, Shannon JR, Furlan R, Robertson RM, Robertson D (1998). Effect of standing on neurohumoral responses and plasma volume in healthy subjects. J Appl Physiol (1985).

